# Acute Bright’s Disease Cured by Jaborandi

**Published:** 1878-11

**Authors:** 


Acute Bright's Disease Cured by Jaborandi.—Prof. J. M. DaCosta, in the *Hospital Gazette*, publishes a clinical lecture, in the course of which he records a case of acute nephritis cured by this drug. The fluid extract of jaborandi was used in drachm doses three times daily. This dose produced excessive diuresis and diaphoresis. At the expiration of five days all symptoms of the disease had disappeared. The woman was left in an extremely prostrated condition, to counteract which *dialyzed* iron was administered both internally and hypodermically.—*The Medical Brief.*

